# Temporal and spatial characteristics of grassland ecosystem service value and its topographic gradient effect in the Yellow River Basin

**DOI:** 10.1371/journal.pone.0278211

**Published:** 2022-12-15

**Authors:** Jie Yang, Baopeng Xie, Shiying Wang, Degang Zhang, Changyu Liu, Erastus Mak-Mensah

**Affiliations:** 1 College of Grassland Science, Gansu Agricultural University, Lanzhou, China; 2 School of Management, Gansu Agricultural University, Lanzhou, China; Soil and Water Resources Institute ELGO-DIMITRA, GREECE

## Abstract

Grassland is the largest terrestrial ecosystem in the Yellow River Basin and an important ecological barrier in the basin. Scientific and accurate estimation of the service value of grassland ecosystems in the Yellow River Basin is of great significance for maintaining the ecological security of the Yellow River Basin. This study was based on Constanza, a method based on the correction of natural grassland ecosystem service value in the Yellow River Basin. This method considers grassland biomass, estimates the ecological service value of each type of grassland item by item, and analyzes their spatial distribution and topographic gradient differentiation characteristics. The results showed that the unit price of soil conservation services provided by the grassland ecosystem in the Yellow River Basin has an advantage (6.2 times) which is the lowest unit price of raw materials. The total value of grassland ecosystem services in the Yellow River Basin is 100.82 × 10^10^ Yuan. Among the various services, the adjustment service has the largest value, accounting for 50.56% of the total value, while the sum of the supply service, support service and cultural service amounted to 49.44%. The high-value areas of grassland ecosystem services are mainly distributed in the upper and lower reaches of the basin, as the low-value areas are located in the Ningxia and Hetao Plains and most parts of the Loess Plateau. The ESV per unit area of grassland in the Yellow River Basin showed a decreasing to increasing trend with the increase in elevation and topographic relief, as the ESV continued to increase with slope increase.

## 1 Introduction

Grassland is the largest terrestrial ecosystem type in China, with a total area of 3.9 × 10^8^ hm^-2^, accounting for 13% of the world’s grassland area and 41.7% of the country’s total land area [1]. The grassland ecosystem not only provides human beings with economic products such as meat, milk and fur, but also has ecological service functions such as carbon fixation and oxygen release, climate regulation, nutrient retention, water conservation and biodiversity protection, and is an important ecological security barrier. It is also an important support for the development of animal husbandry [2]. In the past 50 years, global climate change and unreasonable human development and utilization led to the degradation of 90% of grasslands, resulting in the decline of grassland coverage and net primary productivity, which weakened grassland ecosystem structure and dysfunction [3]. This unbearable pressure has caused serious impact on human health, living environment and quality of life, and has also resulted in a series of social problems [4]. With the deepening of human understanding of grassland ecosystem service functions, more and more studies have been conducted to reflect changes in grassland ecosystem health by explicitly and quantitatively evaluating ecosystem service values.

Since Costanza made a breakthrough in the evaluation of ecosystem service value in the 1990s [5], the upsurge of evaluation of various ecosystem services at home and abroad has immensely promoted the development of ecosystem value evaluation research [5,6]. This includes the concept of ecosystem services [7], functional classification system [8,9], different ecosystems and evaluation indicators [10–12], evaluation methods and research status. Xie et al. formulated the equivalent factor of ecosystem service value in China through the willingness survey method based on China’s national conditions and the results of many scholars such as Costanza [13]. Since then, many scholars have carried out research on the evaluation of ecosystem service value based on the equivalent factor. For example, Zhang et al. used the ecosystem service value table to evaluate the temporal and spatial evolution of the ecosystem service value in the Bosten watershed [14]; Lin et al. evaluated the ecosystem service value of the main stream of the Tarim River, and optimized its land based on the ecosystem service value [15]. Deng et al. used the modified equivalent factor method to calculate the ecosystem service value of 310 counties in the old revolutionary base area along the Long March, considering ESV; they divided and estimated the ecological compensation priority and ecological compensation amount in the area [16]. Gao et al. studied in the Taihu Lake Basin and used the equivalent factor method to calculate ESV, which explored the relationship between ESV and land use intensity and reasons for its changes [17]. However, at present, there are few studies on grassland ecosystem service function and its value evaluation at home and abroad. Because the complexity of grassland ecosystem determines the uncertainty of its function, the ecosystem service value is closely related to its type. Due to the diversity of grassland types, average biomass of grassland in different regions are different, thus the service value of various grassland ecosystems in different regions are quite different. The benchmark unit price of grassland ecosystem services is corrected and accurately estimated using the biomass of different types of grassland in the region and the unit yield and unit price of main grains [18].

The Yellow River Basin is an important ecological barrier and economic zone in China [19]. Grassland is this main land use type in the basin, accounted for about 50% of the total area of the basin. Ecological functions in the basin include soil formation, erosion control, waste disposal, dust retention and biodiversity maintenance [20]. However, due to the frequent human activities and economic development in the Yellow River Basin, the impact on the grassland ecosystem has gradually increased as the speed of grassland retrograde succession has accelerated. Thus, the relationship between human-grass-animals has become unbalanced, and grasslands in a large area have been degraded, through induced sandstorms, desertification and other ecosystems. At present, the research on the grassland ecosystem in the Yellow River Basin mostly focus on degradation status and mechanisms involved [21], the evolution of grassland landscape [22] and the classification of grassland desertification [23]. The service value of grassland ecosystems in the basin is under-studied. Based on this, the unit area equivalent factor method was used in this study to (1) investigate various grassland organisms in the Yellow River Basin to revise the Chinese ecosystem service value equivalent factor proposed by Xie et al. to (2) calculate the grassland ecosystem service value in the Yellow River Basin [24], using GeoDa software for details. The spatial agglomeration characteristics of grassland ESVs in the Yellow River Basin and the topographic gradient effect of grassland ESVs were analyzed, to (1) provide a reliable reference and basis for the formulation of ecological protection planning and management systems and grassland protection policies, and (2) promote the harmonious and sustainable development of man and land.

## 2 Data sources and research methods

### 2.1 Overview of the study area

**Fig 1:**
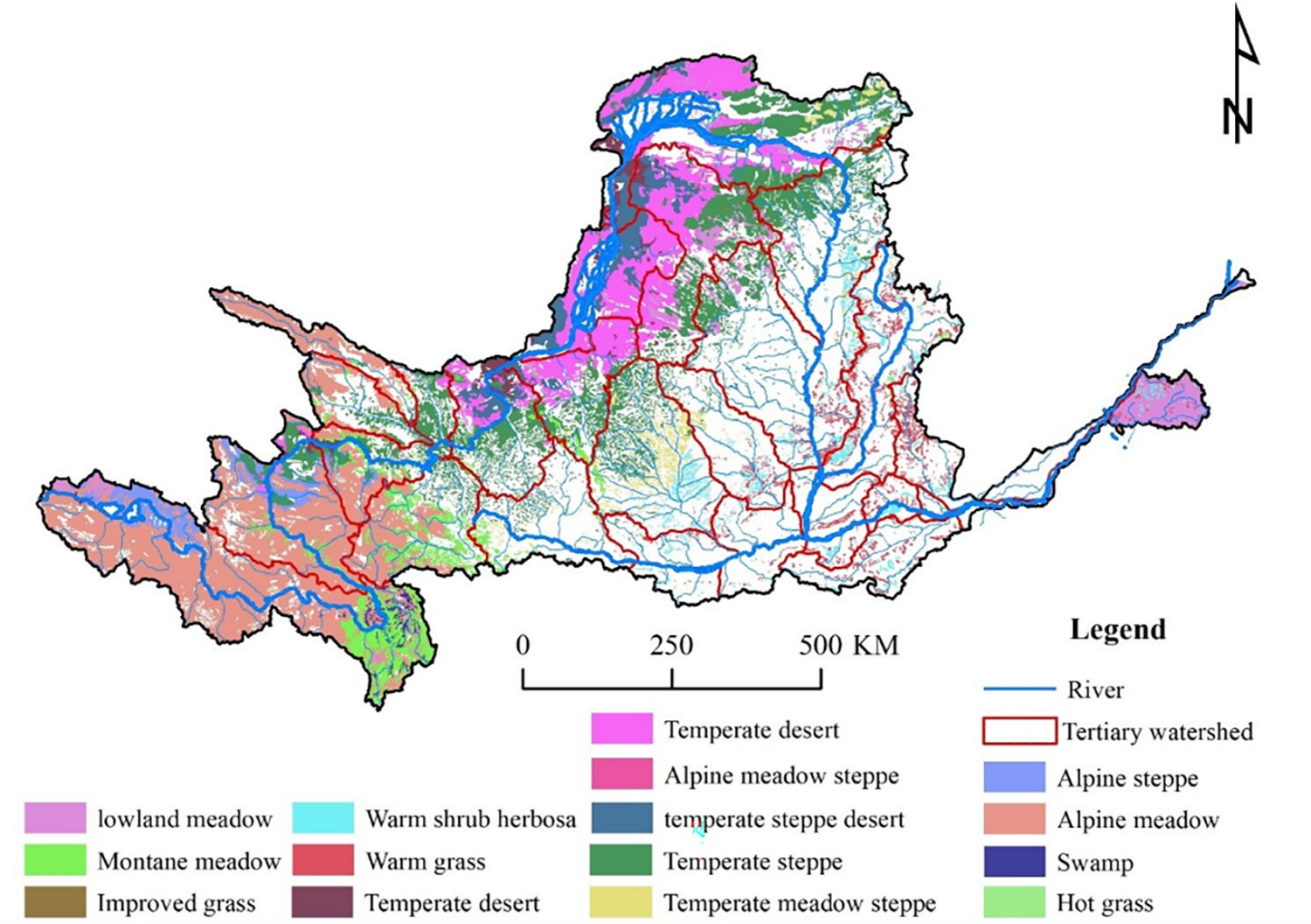
Distribution map of grassland types in the Yellow River Basin.

The Yellow River originates from Yoguzonglie Basin, north of Bayan Har Mountains on the Qinghai-Tibet Plateau, and flows through Qinghai, Sichuan, Gansu, Ningxia, Inner Mongolia, Shanxi, Shaanxi, Henan, and Shandong, into the Bohai Sea in Kenli County, Shandong Province, with a total main stream length of 5464 km and 4480 m for the drop. The Yellow River Basin (YRB) is located between 96° - 119° east longitude and 32° - 42° north latitude (Fig 1), with a length of about 1900 km from east to west, width of about 1100 km from north to south, and a drainage area of 79.5 × 10^4^ km^2^. The upper reach of the Yellow River is located above Hekou Town, with a length of 3472 km and a drainage area of 42.8 × 10^4^ km^2^; while the middle reach is from Hekou Town to Taohuayu, with a length of 1206 km and a drainage area of 34.4 × 10^4^ km^2^; the lower reach is below Taohuayu, with a length of 786 km and a drainage area of only 2.3 × 10^4^ km^2^. The YRB has large territory with many mountains. The height difference between the east and the west is very significant. The topography of each region varies, as well as the climate. The YRB has a large seasonal difference, with annual precipitation ranging from 200 to 650 mm in most parts, and with more than 650 mm in the south of the middle-upper and lower reaches, particularly the northern slope of the Qinling Mountains (700–1000 mm), as it gradually increases from northwest to southeast. Precipitation is unevenly distributed, with a north-south ratio greater than 5. There are 15 different types of grassland in the Yellow River Basin, mainly alpine meadows, temperate grasslands, temperate deserts, and mountain meadows. Distributed in the western part of the Loess Plateau, temperate deserts are mainly distributed in the Ningxia and Hetao Plains, as mountain meadows are widely distributed in the Zoige Plateau.

### 2.2 Data sources and processing

#### 2.2.1 Data sources

Attribute data was adopted from the census data of Chinese grassland resources, the comprehensive survey committee of the Chinese Academy of Sciences (1:1 million grassland resources map of China), and the comprehensive survey committee of the Chinese Academy of Sciences (1:4 million grassland resources map). Statistical data such as grain output and grain prices were taken from the 2019 Statistical Yearbook of Gansu, Qinghai, Shandong and other nine provinces and the "2019 National Agricultural Product Cost and Benefit Data Compilation". The elevation data information such as slope, topographic relief and topographic position index was extracted from the Geospatial Data Cloud (http://www.gscloud.cn/).

#### 2.2.2 Data processing

**Fig 2:**
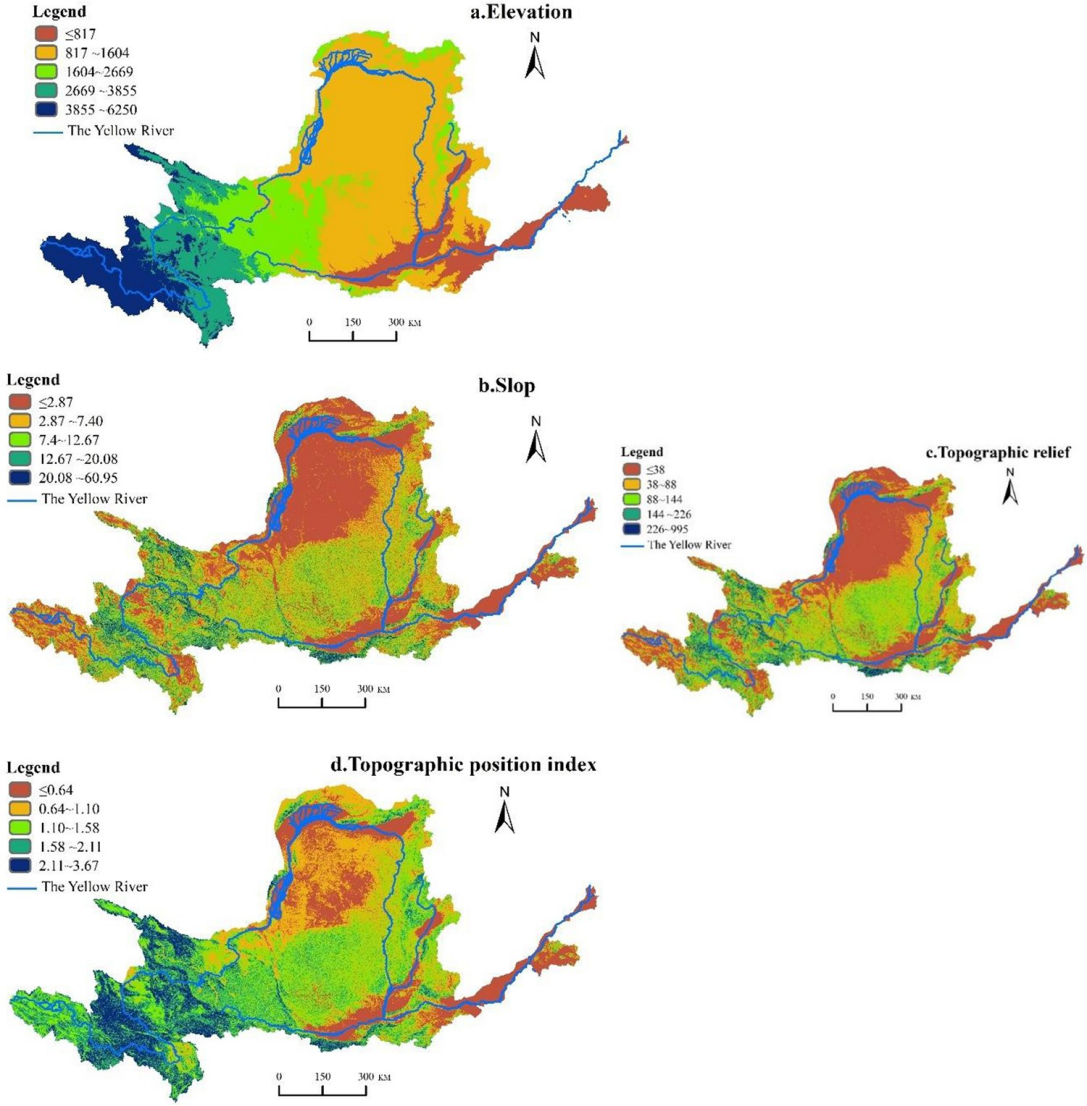
Spatial distribution maps of geomorphological gradients in the Yellow River basin.

ArcGIS 10.2 was used to grade the slope, elevation, topographic position index and topographic relief, in order to avoid uneven area influence on the results. According to existing literature by [25], the quartile method was used to classify topographic which was divided into five levels. According to the divided five-level system, the grassland ecosystem service value of each level was extracted, and finally the average elevation, slope, topographic were obtained through regional statistics. According to their numerical values, they were named as I, II, III, IV and V grades (Table 1), and the spatial distribution is shown in Fig 2.

**Table 1:** Elevation, slope, topographic relief and topographic position index classification standards.

level	Elevation/m	Slope/°	Topographic relief /m	Topographic position index
Ⅰ	<817	0–2.87	<38	<0.64
Ⅱ	817–1604	2.87–7.40	38–88	0.64–1.10
Ⅲ	1604–2669	7.40–12.67	88–144	1.10–1.58
Ⅳ	2669–3855	12.67–20.08	144–226	1.58–2.11
Ⅴ	3855–6250	20.08–60.95	226–955	2.11–3.67

Consequently, the topographic relief degree refers to the height difference between the highest and lowest points in a certain analysis window. This is an important indicator used to quantitatively describe landform shape and divide the landform types which can reflect the macro-regional surface relief characteristics [26]. This indicator is mainly obtained by using the grid neighborhood calculation tool in ArcGIS, and the calculation formula was:

$$Topographic\;relief=A_{max}-A_{min}$$

where A_max_ is the maximum elevation (m) in the analysis window, and A_min_ is the minimum elevation (m) in the analysis window.

The topographic position index (T) is an index that combines the attribute information of elevation and slopes at any point in the analysis space, and can comprehensively reflect the spatial differentiation of topographic conditions. The calculation formula was adapted from [27]:

$$T=\ln\left[\left(\frac{E}{E_{0}}+1)\times (\frac{S}{S_{0}}+1\right)\right]$$

where T is the topographic position index, E and E_0_ are the elevation (m) and average elevation (m) of any grid in the space, respectively, and S and S_0_ are the slope value of any grid in the space (°) and the mean slope value (°), respectively. Generally, the topographic position index of the grid with low elevation and small slope is small, and vice versa.

### 2.3 Research methods

#### 2.3.1 Evaluation method of ecosystem service value

The ecosystem service value equivalent factor is defined as the value of annual natural grain farmland yield with an average national yield of 1 hm^2^. After comprehensive comparative analysis, the value of an ecosystem service value equivalent factor was equal to 1/7 of the market value of the national grain yield in that year [28]. According to Formula (1) and the basic data of the main food crops in the Yellow River Basin, the value of the equivalent factor of an ecosystem service value in the Yellow River Basin was 232.16 USD·hm^-2^·a^-1^

M=(m×n)/7 (1)

where M is the value of an ecosystem service value equivalent factor in the Yellow River Basin; m is the average unit price of the main grains in the Yellow River Basin; and n is the evaluated yield of 1 h m^-2^ of grains in the Yellow River Basin. Using Formula (2) and combining with the Chinese grassland ecosystem service value equivalent table formulated by Xie et al. to calculate the value table of grassland ecosystem services in the Yellow River Basin (*P*_*i*_)

$$P_{i}=M\times d_{i}$$ (2)

where *Pi* is the benchmark unit price of grassland ecosystem services in the Yellow River Basin; M is the value of an equivalent factor of ecosystem services in the Yellow River Basin; i = 1, 2, 3, …, 9, representing food production, raw materials, water Conservation, soil conservation, waste disposal, gas regulation, climate regulation, biodiversity conservation, entertainment and ecosystem service functions.

Eq (3) and the biomass of different grassland types in the Yellow River Basin were used to calculate the ecosystem service value of different grassland types in the Yellow River Basin.

Pij=(bjB)×Pi (3)

where P_ij_ is the ecosystem service value per unit area of grassland in the Yellow River Basin, bj is the biomass of the j-type grassland; B is the average biomass per unit area of the grassland in the whole region; P_i_ is the benchmark unit price of the grassland ecosystem service function in the Yellow River Basin. Letter j = 1, 2, 3 …,15, respectively, represent temperate meadow, warm shrub and grass, temperate grassland, warm grass, improved grassland, mountain meadow, lowland meadow, swamp, alpine meadow, temperate desert, temperate Grassland, hot grass, temperate desert, alpine grassland, and alpine meadow grassland.

The total value of grassland ecosystem services in the Yellow River Basin and in various grassland areas was calculated using Formula (4).


$$T_{ij}=P_{ij}\times A_{j}$$ (4)

Where T_ij_ is the total service value of grassland ecosystem in the Yellow River Basin; P_ij_ is the ecosystem service value per unit area of grassland in the Yellow River Basin; and A_j_ is the area of different grassland types in the Yellow River Basin.

#### 2.3.2 Exploratory spatial data analysis

Exploratory Spatial Data Analysis (ESDA) is the collection of spatial data analysis techniques and methods which could be used to describe the spatial distribution of data and through visual representation to explore spatial data structure, and to reveal natural phenomena and the spatial interaction mechanism between them [29]. Spatial autocorrelation refers to the potential interdependence between the observed data of some variables in the same distribution area. Spatial autocorrelation statistics refers to the measure of basic geographic data properties at a certain location and at other locations and the degree of interdependence between them. This includes global and local autocorrelation analysis. Global autocorrelation analysis is used to describe the spatial characteristics of the study area and to test whether the study objects have spatial agglomeration [30]:

$$Moran^{\prime }s\;I=\frac{\sum_{i=i}^{n}\sum_{j=1}^{n}W_{ij}(Y_{i}-\bar{Y})(Y_{j}-\bar{Y})}{\sum_{i=1}^{n}\sum_{j=1}^{n}W_{ij}}\times \frac{n}{\sum_{i=1}^{n}Y_{i}}$$ (5)

where n is the total number of study areas, Y_i_ is the observation value of the i-th area, W_ij_ is the spatial weight matrix, and *Moran′s I* value range was [–1, 1]. When Moran’s I > 0, similar observations tend to be spatial agglomeration. When Moran’s I < 0, similar observations tend to be scattered, and when Moran’s I = 0, there is no spatial dependence.

Local autocorrelation analysis could be used to refine and analyze local features and changes in space. The most commonly used are Moran scatter plot and LISA cluster plot for visual analysis. The scatter plot is divided into four quadrants, and the first quadrant is high and high aggregation (HH), where the ESV of both the surface area and the surrounding area is high. The second quadrant is low-high agglomeration (LH), where areas with lower surface ESV are surrounded by areas with higher ESV. The third quadrant is low and low agglomeration (LL), where the ESV of the region and surrounding regions are low. The fourth quadrant is high and low clustering (HL), where the areas with higher ESV are surrounded by areas with lower ESV. The first and third quadrants are typical areas, while the second and fourth quadrants are atypical areas. The formula for calculating the Local Moran’s I index is [31]:

$$I_{i}=z_{i}\sum_{j=1}^{n}w_{ij}\;z_{j}$$ (6)

where z_i_ and z_j_ are the normalized observation vectors of regions i and j, w_ij_ is the spatial weight matrix, and n is the total number of study regions.

## 3 Results

### 3.1 Benchmark unit price of grassland ecosystem services in the Yellow River Basin

The services provided by grassland ecology in the Yellow River Basin (Table 2), the benchmark unit prices of soil conservation, biodiversity conservation, and climate regulation are relatively high, which are 5200.38 Yuan hm^-2^, 4341.39 Yuan hm^-2^, and 3621.69 Yuan hm^-2^ respectively. Among all services, especialy in food production, the unit price of raw materials and raw materials is the lowest at 998.29 Yuan hm^-2^ and 835.77 Yuan hm^-2^ respectively, the sum of which is not as good as the value of entertainment. The values of water conservation, waste disposal and gas regulation are not much different, but they are all higher than entertainment, which are 3482.4 Yuan hm^-2^, 3528.83 Yuan hm^-2^ and 3064.51 Yuan hm^-2^ respectively. The value of soil conservation with the highest unit price is the lowest with 6.2 times the unit price of raw materials. This shows that the value generated by the soil conservation services provided by the grassland ecosystem in the Yellow River Basin is absolutely dominant.

**Table 2:** Benchmark unit price of various types of grassland ecosystem services in the Yellow River Basin. Unit: Yuan/hm^2^.

Ecosystem service function	Food production	Raw materials	water conservation	Soil conservation	waste disposal	Gas regulation	climate regulation	Biodiversity conservation	Entertainment	Total
Temperate meadow steppe	1139.34	953.86	3974.44	5935.16	4027.43	3497.50	4133.41	4954.80	2305.17	30921.12
Warm shrub herbosa	1742.47	1458.81	6078.39	9077.06	6159.43	5348.98	6321.52	7577.72	3525.46	47289.84
Temperate steppe	498.78	417.58	1739.92	2598.28	1763.12	1531.13	1809.51	2169.10	1009.15	13536.56
Warm grass	1606.46	1344.95	5603.94	8368.55	5678.66	4931.47	5828.10	6986.24	3250.28	43598.64
Improved grass	773.94	647.95	2699.79	4031.69	2735.79	2375.82	2807.78	3365.74	1565.88	21004.37
Montane meadow	1830.53	1532.53	6385.56	9535.77	6470.70	5619.29	6640.98	7960.66	3703.62	49679.64
Lowland meadow	819.43	686.03	2858.48	4268.66	2896.59	2515.46	2972.82	3563.57	1657.92	22238.97
Swamp	1681.13	1407.46	5864.41	8757.51	5942.60	5160.68	6098.98	7310.96	3401.36	45625.09
Alpine meadow	1337.07	1119.41	4664.19	6965.19	4726.38	4104.49	4850.76	5814.69	2705.23	36287.38
Temperate desert	163.58	136.95	570.63	852.14	578.24	502.15	593.46	711.39	330.97	4439.50
Temperate steppe desert	90.97	76.16	317.33	473.88	321.56	279.25	330.02	395.61	184.05	2468.84
Hot grass	2384.70	1996.50	8318.73	12422.64	8429.65	7320.48	8651.48	10370.68	4824.86	64719.73
Temperate desert steppe	169.52	141.92	591.35	883.09	599.24	520.39	615.01	737.22	342.98	4600.72
Alpine steppe	596.31	499.24	2080.16	3106.37	2107.90	1830.54	2163.37	2593.27	1206.49	16183.64
Alpine meadow steppe	140.09	117.29	488.69	729.78	495.21	430.05	508.24	609.24	283.44	3802.04

From the perspective of different types of grassland, ecosystem service values are also different. Among them, the service value per unit area of thermal grassland is the highest, with 64719 Yuan hm^-2^; the ecological service value per unit area of warm desert steppe is the lowest, with 4600.72 Yuan hm^-2^. The ecosystem service value of different grassland types per unit area are in the order of thermal grassland, mountain meadow, warm shrub and grass, swamp, alpine meadow, temperate meadow grassland, lowland meadow, improved grassland, alpine grassland, temperate grassland Steppe, temperate desert steppe, temperate desert, alpine meadow steppe, and temperate steppe desert.

### 3.2 The total value of grassland ecosystem services in the Yellow River Basin

According to the benchmark unit price of grassland ecosystem combined with the area of different types of grassland, the service value of various grassland ecosystems in the Yellow River Basin was calculated (Table 3). The total value of grassland ecosystem services in the Yellow River Basin was 100.82 × 10^10^ Yuan. Among the various services, the value of supply services (food production and raw materials) was 68.25 × 10^9^ Yuan, amounting to 6.77% of the total value of grassland ecosystem services; regulation services (including climate regulation, gas disposal regulation, waste disposal, water conservation) value was 50.97 × 10^10^ Yuan, accounting for 50.56% of the total value of grassland ecosystem services; support services (soil conservation and biodiversity conservation) value was 35.50 × 10^10^ Yuan, accounting for 50.56% of the total value of grassland ecosystem services, the value of the remaining cultural services was 7.51 × 10^10^ Yuan, amounting to 7.45%.

**Table 3:** Total value of various grassland ecosystem services in the Yellow River Basin. Unit: × 10 ^8^ Yuan.

Ecosystem service function	Food production	Raw materials	water conservation	Soil conservation	waste disposal	Gas regulation	climate regulation	Biodiversity conservation	Entertainment	Total
temperate meadow steppe	9.042	7.570	31.543	47.105	31.964	27.758	32.805	39.324	18.295	245.407
Warm shrub herbosa	22.624	18.941	78.921	117.855	79.973	69.450	82.077	98.388	45.774	614.002
Temperate steppe	45.708	38.267	159.446	238.105	161.571	140.312	165.823	198.775	92.478	1240.486
warm grass	26.899	22.520	93.835	140.126	95.086	82.574	97.588	116.980	54.424	730.033
improved grass	0.316	0.265	1.103	1.648	1.118	0.971	1.147	1.375	0.640	8.583
Montane meadow	53.387	44.696	186.234	278.109	188.717	163.885	193.683	232.171	108.015	1448.897
Lowland meadow	21.670	18.142	75.593	112.885	76.601	66.522	78.616	94.239	43.844	588.112
swamp	4.489	3.758	15.658	23.383	15.867	13.779	16.284	19.520	9.082	121.820
Alpine meadow	166.335	139.257	580.239	866.490	587.976	510.610	603.449	723.365	336.539	4514.259
Temperate desert	1.314	1.100	4.585	6.847	4.646	4.035	4.769	5.716	2.659	35.672
temperate steppe desert	1.847	1.546	6.442	9.620	6.528	5.669	6.700	8.031	3.736	50.118
hot grass	0.007	0.006	0.025	0.037	0.025	0.022	0.026	0.031	0.014	0.193
Temperate desert steppe	10.083	8.441	35.172	52.523	35.641	30.951	36.579	43.848	20.400	273.637
Alpine steppe	7.757	6.495	27.061	40.411	27.422	23.814	28.143	33.736	15.695	210.533
Alpine meadow steppe	0.018	0.015	0.062	0.093	0.063	0.055	0.064	0.077	0.036	0.482

The contribution rate of different types of grasslands to the ecosystem service value was also different, among which the alpine meadow has the highest ecosystem service value, which was 45.12 × 10^10^ Yuan, and the contribution rate was 44.75%, followed by the mountain meadow with ecosystem service value of 14.48 × 10^10^ Yuan, with a contribution rate of 14.36%. The ecosystem service value of temperate grassland was 12.40 × 10^10^ Yuan, accounting for approximately 12.30%. The ecosystem service values of warm grass, warm shrub-grass and lowland meadow were 7.30 × 10 ^10^ Yuan, 6.14 × 10 ^10^ Yuan and 5.88 × 10 ^10^ Yuan, respectively. The ecosystem service value of alpine steppe, temperate meadow steppe and temperate desert steppe were almost the same, all of which was greater than 2.5 × 10^10^ Yuan. The thermogenic grass species had the lowest ecosystem service value, which was 0.19 × 10^8^ Yuan.

### 3.3 Spatial distribution of ecosystem service value

**Fig 3:**
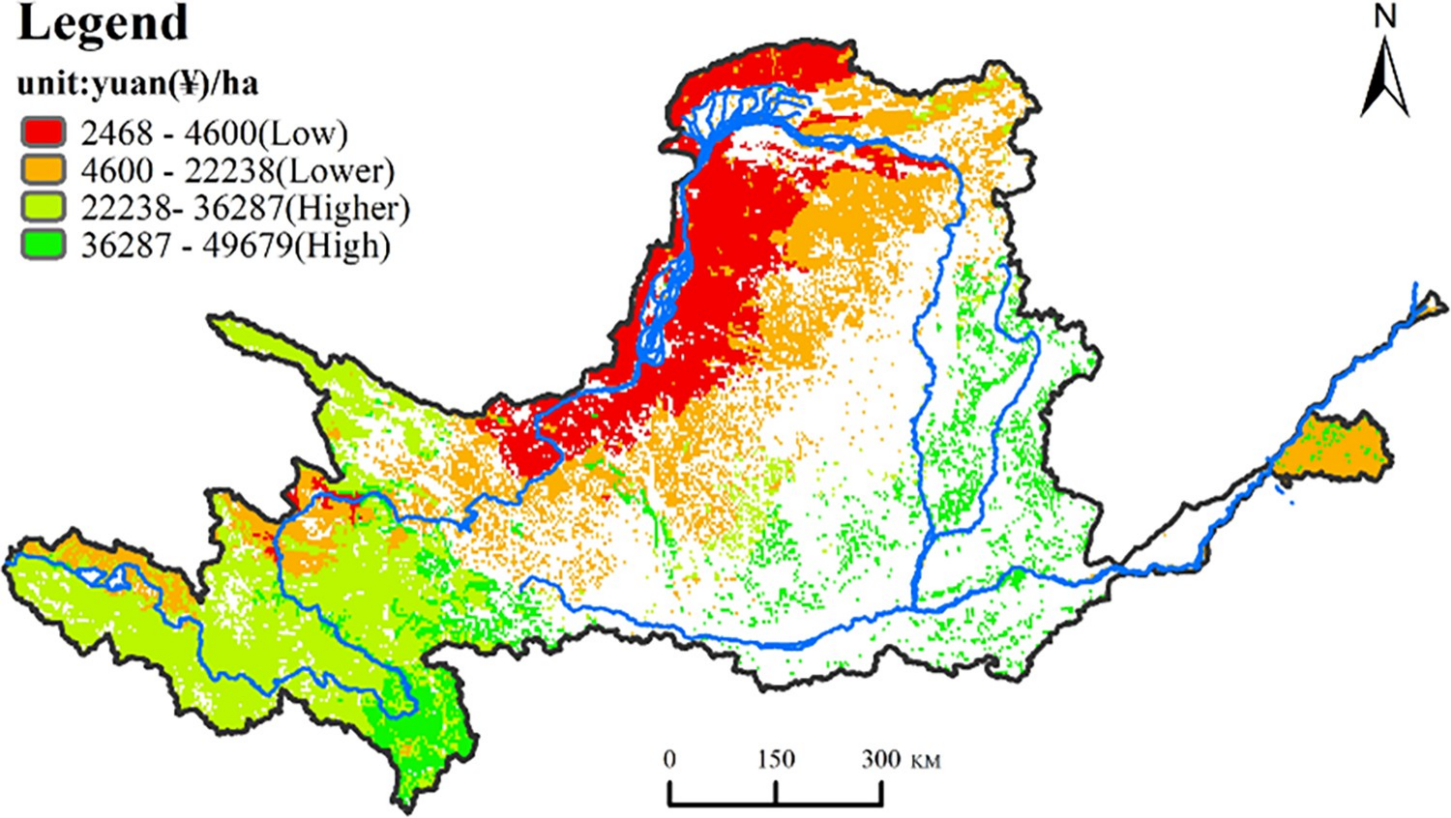
Spatial distribution of benchmark unit price of grassland ecosystem services in the Yellow River Basin.

As shown in Fig 3, the natural breakpoint method is used to divide its values into 4 categories from large to small, namely higher-value area, high-value area, low-value area, and lower-value area. Overall, the service value of grassland ecosystems in the Yellow River Basin showed a spatial pattern of high and low in southwest and northwest, respectively, and the spatial distribution of grassland ESV and grassland types had a strong spatial consistency. The high-value areas are mainly distributed in the Zoige Plateau, the source area of the Yellow River, and sporadically distributed in the Qinghai-Tibet Plateau and lower Taihang Mountains. The main grassland types in these areas are mountain meadows with high vegetation coverage. The higher-value area has a larger area and is concentrated in the Qinghai- Tibet Plateau in the upper reaches of the Yellow River, forming an obvious geographical unit. The grassland types in this area are mainly alpine meadows, with few temperate meadows and grasslands. The lower value area is mainly distributed in the western area of the Loess Plateau, and the distributed grassland types include alpine steppe, temperate steppe and lowland meadow. The low-value areas are mainly distributed in the middle reaches of the Yellow River Basin, the Ningxia and Hetao Plains and northern Loess Plateau. The main types of grasslands are temperate desert steppe, temperate steppe desert and temperate desert.

**Fig 4:**
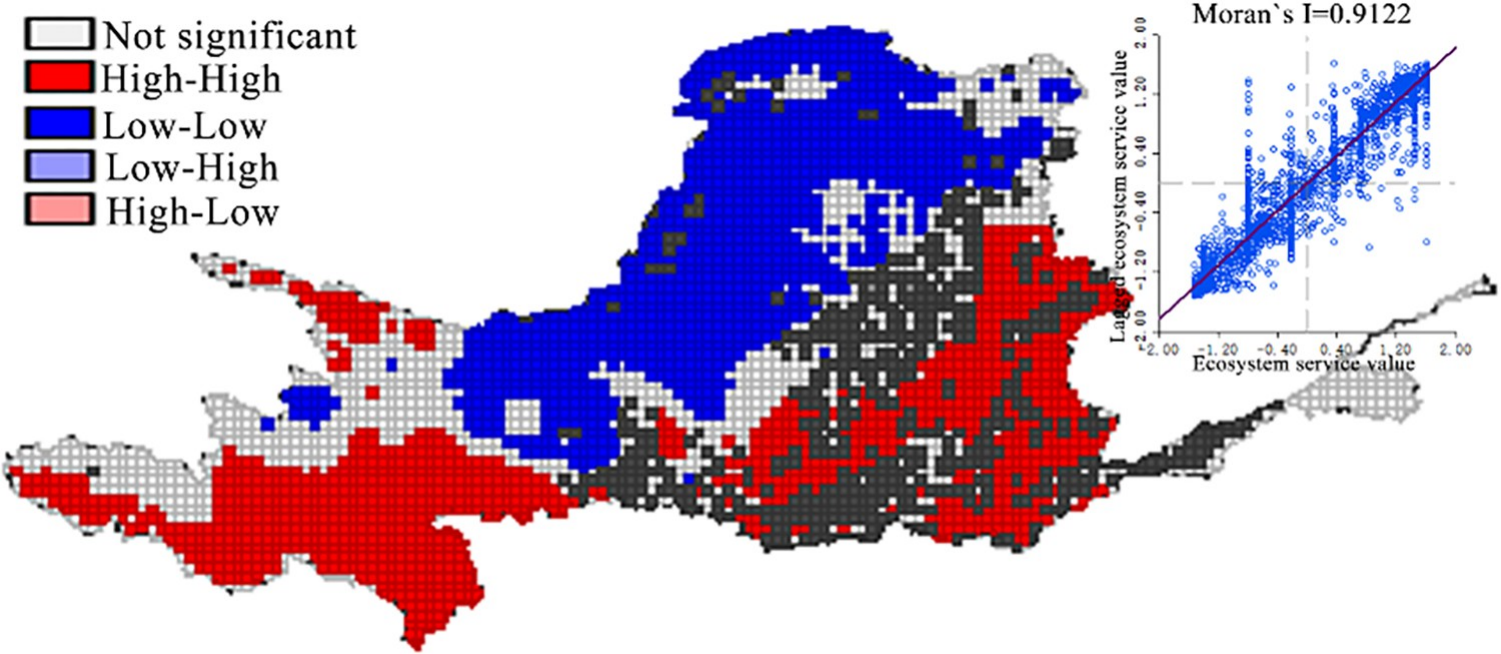
Spatial autocorrelation distribution of grassland ecosystem service value in the Yellow River Basin.

In order to further reveal the spatial distribution characteristics of grassland ecosystem service value in the Yellow River Basin, a univariate spatial autocorrelation analysis was conducted using the benchmark unit price of grassland ecosystem services as a variable. The analysis showed that grassland ESV had significant spatial aggregation characteristics, and most of the grids are in high-high and low-low aggregation areas, indicating that grassland ESV have high and low-value aggregation characteristics in space. The LISA agglomeration map was further used to judge the type of local spatial autocorrelation and its significance level. When *P* = 0.05, the local spatial autocorrelation of grassland ESV is shown in Fig 4. The characteristic of grassland ESV accumulation in the Yellow River Basin was obvious. The higher and high agglomeration areas are distributed in the upper and lower reaches of the Yellow River, and the lower and low agglomeration areas are distributed in the Hetao and Ningxia Plains and most parts of the Loess Plateau. There are some small-scale "flower arrangement" agglomeration areas, such as higher and high agglomeration areas in the Qilian Mountains.

### 3.4 Topographic gradient differentiation characteristics of grassland ecosystem service value in the Yellow River Basin

**Fig 5:**
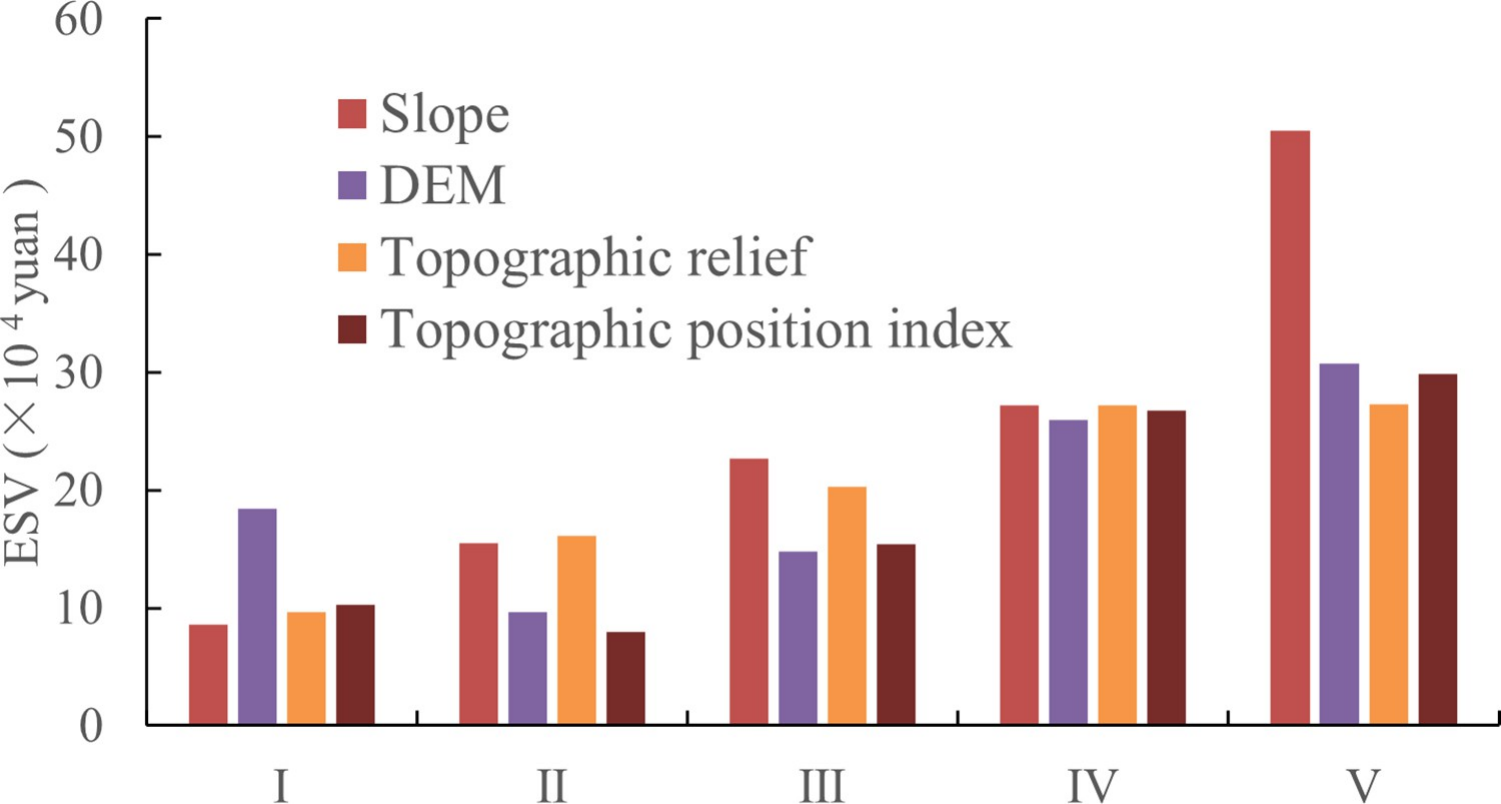
Variation of ecosystem service value with topographic gradient.

In order to ensure the comparability of ESVs on grid units, a fishnet map was generated, and the average values of elevation, slope, topographic relief and topographic position index at the center point of the grid were extracted, then average values of grassland ESVs at all levels of topographic gradient were classified and drawn. From the figure, it can be seen that the elevation gradient effect of ESV per unit area of grassland in the Yellow River Basin was significant. With the increase in elevation, ESV shows a decreasing-rising trend. ESV was the lowest at level II elevation, while at level V ESV was the highest. From topographical point of view, the gradient zone is mainly distributed with alpine meadow grasslands, with few alpine types of grassland. The vegetation coverage could span from 70 to 90%, which contributes greatly to the service function of grassland ecosystem, thus leading to higher ESV. The slope gradient characteristics of ESV were obvious. As slope increases, ESV continues to rise. First, it could be related to the slope area size at all levels in the Yellow River Basin and second, due to the particularity of study area location. From the figure, it can be observed that when the slope grade was V grade, the grassland ESV was the highest, with small slope grade area distributed in the entire Yellow River basin, but mostly concentrated in the upper Qinghai-Tibet Plateau. The range of topographic relief in the Yellow River Basin was between 0 and 955 meters. The grassland ESV in the Yellow River Basin increases first and then decreases with topographic relief increase. Grassland ESV is the lowest at level I and highest at level VI; the maximum value of the topographic position index is 3.66, which indicates that the local area has the characteristics of large slope and high elevation, with minimum topographic position index of 0.18. The grassland ESV first decreased and then increased with increase in topographic position index. Grassland ESV was the highest when topographic position index was V (Fig 5).

**Fig 6:**
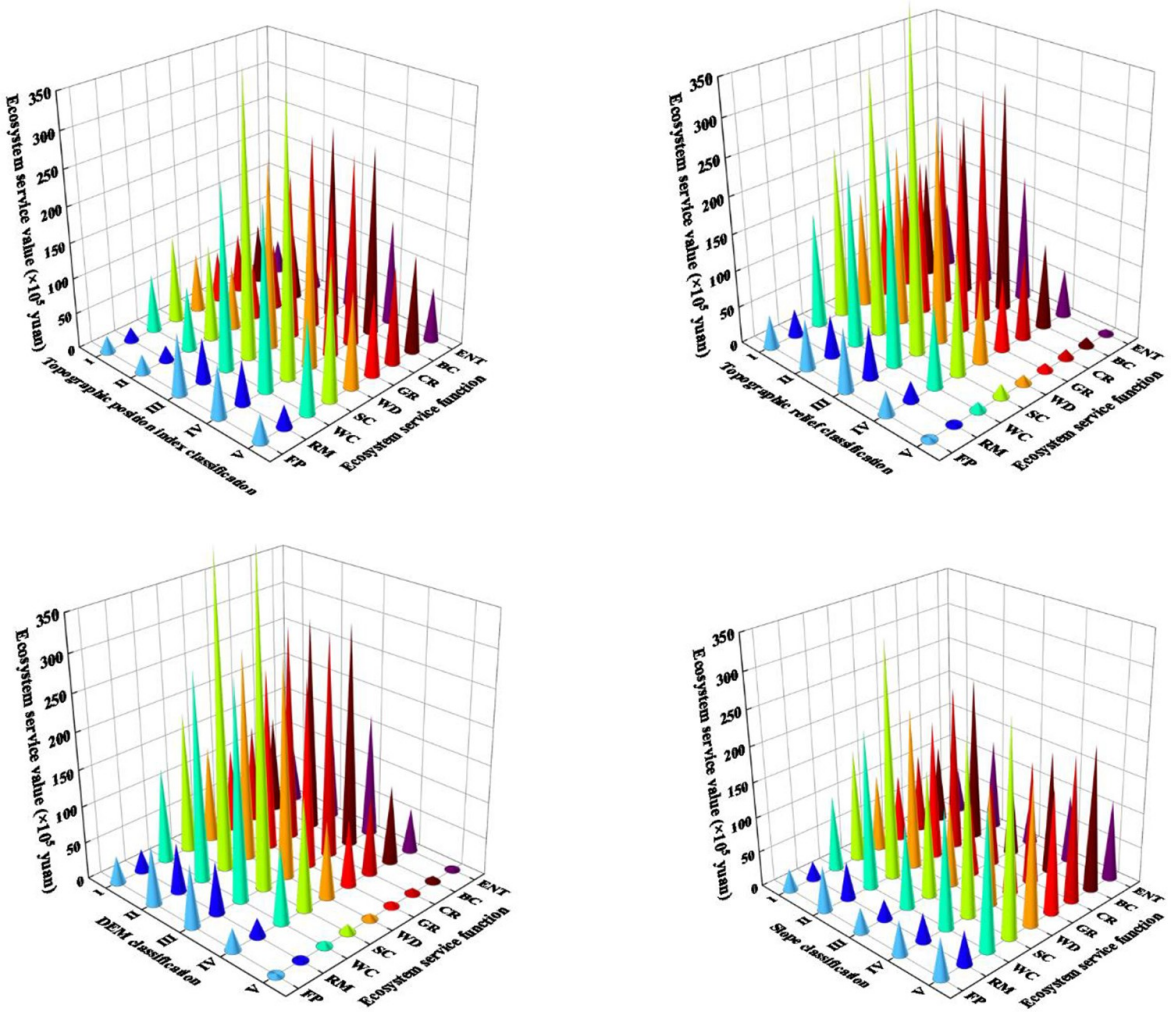
Changes in the value of various ecosystem services with the topographic gradient.

The ESVs of various grassland ecosystem services in the Yellow River Basin have obvious topographic gradient characteristics, as shown in Fig 6. With the continuous increase of in elevation, the change rules of the service values of various grassland ecosystems are basically the same, with an increase first and then a decrease. The value of each ecosystem service is was at the highest at levels II and III, and the value of each ecosystem service is at the lowest at level V, which is was related to the area of the DEM area level, level and secondly due to the particularity of the location of the study area location. Among ecosystem services, Soil conservation hasd the highest value, followed by climate regulation, and food production and raw materials which had the lowest value. The service value of each ecosystem showed a change pattern of “rising-falling-rising” with slope increase. On the whole, the service value of each ecosystem was the largest when the slope was grade II, and the lowest when the slope was grade I; the soil conservation value was the largest, while raw materials had the lowest value. The service value of various ecosystems generally showed a trend of first rising and then decreasing with the increase of the topographic position index and topographic relief degree. The value of each ecosystem service was the largest when the topographic position index and topographic relief degree was grade III, and the topographic position index was I. When the value of soil conservation was the largest, and that of the raw materials was the lowest, the topographic position index and topographic reliefs were V (lowest), the ecosystem service and slope and DEM results each were similar.

## 4 Discussion

As a bridge and link of coupling and interaction between the natural environment and human well-being, ecosystem services have become an important topic in the field of human-land relations [32]. The grassland ecosystem service value analysis transforms ecological problems into easy-to-understand indicators, which estimate the incremental value or marginal value of grassland ecosystem services according to the principles of ecological economics, so as to clarify the potential value of grassland ecosystem services. According to [33], in order to find problems and improve people’s correct understanding of the importance of grassland ecosystem services, the potential dangers caused by over-utilization [34], needs to be analyzed to reveal the multiple standards of ecological, economic and social benefits. The economic development and ecological protection planning and management decisions are very important to maintain and protect the maximum natural capital of grassland, scientific and rational utilization and protection of grassland resources. Topographic is an important basis for affecting the distribution, structure and function of ecosystem services. Elevation, slope, topographic relief and topographic position index were selected to explore the changes in the value of grassland ecosystem services in the Yellow River Basin on these four topographic elements. The cross-sectional analysis of the system service value in vertical space provides scientific reference for the coordinated development of grassland in the basin and the optimization of ecosystem services. The assessment of grassland ecosystem service value requires data availability and accuracy. With continuous evolution of the relationship between humans and the natural environment, the equivalent factor table needs to adapt to the dynamic update of the times; considering the locality of grassland ecosystem service value research, more time-sensitive Local and local ecological indicators and high-precision remote sensing data should be incorporated into the evaluation system; the grassland ecosystem service evaluation model can fully consider ecological processes and will become the future direction of grassland value evaluation.

This research conducted in the Yellow River Basin, has important strategic significance, as the research objective, uses the "Ecosystem Service Value Equivalent per Unit Area of Ecosystem in China" proposed by Xie et al. The estimated value of grassland ecosystem services in the Yellow River Basin was 100.82 × 10^10^ Yuan, indicating that the value of grassland ecosystem services in the Yellow River Basin was huge. Among the services provided by grassland ecology, soil conservation has the greatest service value, indicating that grasslands have the strongest ability to maintain water and soil, and is similar to results obtained by Pan et al. [18], who revealed that the value of grassland ecosystem services was related to the special geography. Meanwhile, the value of food production and raw materials are low, and had negative impact on the healthy development of the regional economy. In the future, corresponding protection measures should be formulated in accordance with the geographical distribution of the service value of the grassland ecosystem service system, such as construction in cities with bare lands. In the process of economic development in concentrated land area, cultivated land, and water areas, wetlands should be strictly protected; ecological lands such as forest land and grassland in mountainous areas should be prevented from occurring in the development process (soil erosion). Among different grassland types, the alpine meadow has the highest (up to 44.75%) contribution rate to the ecosystem service value, indicating that the alpine meadow is the most important ecological service value producing grassland in the Yellow River Basin, and has an important role as an ecological security barrier.

The grassland ecosystem service value has an obvious gradient effect on the terrain. The grassland ecosystem service value first decreases and then increases with the DEM and topographic position index. In the DEM, because the DEM in the basin is high in the west and low in the east, the benchmark unit price for Tall grasslands are mostly distributed in areas with higher DEM, so the ESV of grassland is the highest at level Ⅴ. Due to the difference in human activity intensity on different slope grades, the related ecosystem service functions will also change accordingly, and the grassland ESV will continue to increase with slope increase. Since construction lands are cover a vast part of the ecosystem compared to grassland areas which are relatively small, grassland ESV is low. With increase in slope, natural conditions such as water and heat are moderate, when influence of human activities is gradually reduced, and the grassland vegetation grows better, leading to higher grassland ESV. The spatial pattern of topographic relief and slope in the watershed is similar, so grassland ESV variation characteristics on topographic relief are similar to those on slope gradient. The distribution characteristics of grassland ESV on the topographic gradient are the result of the comprehensive influence of various factors, mainly including natural, socio-economic and ecological policy factors. It has an important influence and preliminarily determines the grassland ecological ESV level in the study area where socioeconomic factors are mainly reflected in human activity intensity such as farming and construction. By changing the natural landscape of the region, the structure, process and function of the ecosystem changes [35].

In this study, keeping the ecosystem value coefficient consistent in space, more objectively and truly reflect the relative spatial difference of grassland ecosystem service value [36]. The spatial autocorrelation and gradient analysis were used to preliminarily study the Yellow River Basin grassland ESV topographic gradient. This has the significance to reflect the spatial differences of ecosystem services and different service values. Nevertheless, species composition, community structure, species richness and other attributes in the grassland ecosystem will affect the grassland biomass. This emanated since ecosystem value depends not only on the factors mentioned above factors, but also on factors such as the location of the ecosystem in space. Ecosystem services are intrinsically linked, but related socioeconomic factors cannot be ignored. This study used data on grassland types to predict ESV, but due to the different driving factors of different grassland types, these factors are not considered, so there is a lack of analysis for each grassland type. A type of quantitative research to reveal the ecological processes and response mechanisms of this condition is one of key research directions in the future. Secondly, this paper only discusses the spatial heterogeneity of grassland ecosystem service value, and does not further discuss the trade-off and synergistic relationship between various services. In the future, an ecosystem service evaluation model should be considered for systematic analysis.

## 5 Conclusion

Conservation services provided by the grassland ecosystem in the Yellow River Basin have advantages, which include lowest unit price of raw materials. Furthermore, the service value per unit area of thermal grassland was the highest, while the ecological service value per unit area of temperate desert steppe was the lowest. The total grassland ecosystem services value in the Yellow River Basin was 100.82×10^10^ Yuan. Among the various services, the adjustment service had the largest value, and the sum of the supply service, support service and cultural service were relatively high Alpine meadows had the highest ecosystem service value followed by mountain meadows. The grassland ecosystem services value in the Yellow River Basin revealed a spatial pattern of high in the southwest and low in the northwest. This resulted in a spatial distribution of grassland ESV and grassland types had strong spatial consistency. The grassland ESV per unit area in the Yellow River Basin showed a relatively obvious topographic gradient difference, and it showed a decreasing to increasing trend with elevation and topographic relief increase. The high indicates the dynamic of first falling and then increasing.

## Supporting information

S1 File (ZIP)

S2 File (ZIP)

## References

[1] Zhao TQ, Ouyang ZY, Zheng H, Wang XK, Miao H. Ecosystem services and their valuation of China grassland. Acta Ecologica Sinica, 2004, 24(6): 1101–1110.

[2] The Ministry of Agriculture Animal Husbandry and Veterinary Department of the People’s Republic of China, The National Animal Husbandry and Veterinary Station. Grassland Resources of China. Beijing: China Science and Technology Publishing House, 1996.

[3] Liu XY, Mu YT. Research progress in the ecosystem services function and value of grasslans. Acta Prataculturae Sinica. 2012, 21(6): 286–295.

[4] Zhang MF. Strengthening grassland protection is an important measure to maintain national ecological security. Acta Prataculturae Sinica, 2006, 23(6): 107–109.

[5] Costanza R, d’Arge R, de Groot R, et al. The value of the world’s ecosystem services and natural capital. Nature, 1997, 387: 253–260.

[6] Zhang ZM, Liu JG. Progress in the valuation of ecosystem services. Acta Scientiae Circumstantiae, 2011, 31(9): 1835–1842.

[7] Sun B, Wang BY, Feng J, Jiang JC, Pan DR, Wang H, et al. Monitoring models of the grass yield for grassland in Gansu Province. Pratacultural Science, 2015, 32(12): 1988–1996.

[8] Zhao TQ, Ouyang ZY, Zheng H, Wang XK, Miao H. Forest ecosystem services and their valuation in China. Journal of Natural Resources, 2004, 19(4): 480–491.

[9] Zhao TQ, Ouyang ZY, Wang XK, Miao H, WeiYC. Ecosystem services and their valuation of terrestrial surface watersystem in China. Journal of Natural Resources, 2003, 18(4): 443–452.

[10] Zhang LL, Gong J, Zhang Y. A review of ecosystem services: A bibliometric analysis based on web of science. Acta Ecologica Sinica, 2016, 36(18): 5967–5977.

[11] Li L, Wang XY, Luo L, Ji XY, Zhao Y. A systematic review on the methods of ecosystem services value assessment. Chinese Journal of Ecology, 2018, 37(4): 1233–1245.

[12] Xu WP, Kang WX, He JN. Analysis of the value of ecosystem services in the Dongting Lake area. Acta Prataculturae Sinica, 2016, 25(1): 217–229.

[13] Xie GD, Zhen NL, Lu CX, Xiao Y, Chen C. Expert knowledge based valuation method of ecosystemservices in China. Journal of Natural Resources, 2008, 23(5): 911–919.

[14] Zhang M, Dilinuer A, Spatiotemporal evolution and trade-off synergy of ecosystem service value in Bosten Lake Basin. Journal of Hydroecology, 10.15928/j.1674-075.202104060094.

[15] Lin J, Zhao CY, Ma XF, Shi FZ, Wu SX, Zhu J. Optimization of land use structure based on ecosystem service value in the mainstream of Tarim river,. Arid Zone Research, https://kns.cnki.net/kcms/detail/65.1095.X.20210611.1741.006.html.

[16] Deng YX, Hou MY, Jia L, Wang YQ, Zhang X, Yao SB. Ecological compensation strategy of the old revolutionary base areas along the route of Long March based on ecosystem service value evaluation. Chinese Journal of Applied Ecology, 10.13287/j.1001-9332.202201.019. 35224938

[17] Xin G, Shen JQ, He WJ, Zhao X, Li ZC, Hu WF, et al. Spatial-temporal analysis of ecosystem services value and research on ecological compensation in Taihu Lake Basin of Jiangsu Province in China from 2005 to 2018. Journal of Cleaner Production, 2021, 317, 128241.

[18] Pan DR, Yan HW, Han TH, Sun B, Jang JC, Liu XN, et al. Evaluation of the service function value of grassland ecosystems in Gansu Province using the equivalence factor method. Pratacultural Science, 2021, 38 (9): 1860–1868.

[19] Xu H, Shi N, Wu L L, et al. High-quality development level and its spatiotemporal changes in the Yellow River Basin[J]. Resources Science. 2020, 42(1): 115–126.

[20] White R, Murray S, Rohweder M. Pilot Analysis of Global Ecosystems: Grassland Ecosystems. World Resources Institute, Washington DC.2000.

[21] Chen WY, Zheng HP, Qi DC, et al. Species diversity changes in converse successions of grassland in important eco-function area in the upper reaches of the Yellow River[J]. Chinese Journal of Grassland. 2007, 29 (6): 6–11.

[22] Liu B, Yang H, Xu F, et al. Analysis of grassland landscape evolution in the Yellow River Basin based on MODIS Data [J]. Journal of Xinyang Normal University (Natural Science Edition). 2018, 31(4): 586–591.

[23] Zheng QZ, Xu XY, Liu XY, et al. Classification and control of grassland desertification in Maqu of the upper Yellow River[J]. Technology of Soil and Water Conservation, 2012(05):1.

[24] Xie GD, Zhang YL, Lu CX, Zheng D, Cheng A K. Study on valuation of rangeland ecosystem services of China. Journal of Natural Resources, 2001, 16(1): 47–5.

[25] Xu CX, Gong J, Li Y, et al. Distribution characteristics of typical ecosystem services in Bailong River Basin in Gansu based on topographic gradient [J]. Acta Ecologica Sinica, 2020, 40(13): 4291–4301.

[26] Zhang L. Research on the classification of topographic morphology based on topographic relief [D]. Hebei Normal University, 2009.

[27] Mugagga F., Kakembo V., Buyinza M., Land use changes on the slopes of Mount Elgon and the implications for the occurrence of landslides, CATENA, Volume 90, 2012, Pages 39–46, ISSN 0341-8162.

[28] Xie GD, Zhang YL, Lu CX, ZHENG D, CHENG S K. Study on valuation of rangeland ecosystem services of China. Journal of Natural Resources, 2001, 16(1): 47–53.

[29] Anselin L., Cho WKT, 2002. Spatial effects and ecological inference. Polit. Anal. 10(3), 276–297. 10.1093/pan/10.3.276.

[30] Symanzik J., 2013. Exploratory spatial data analysis. Handbook of Regional Science. Springer, pp. 1295–2310.

[31] Yang Q, Chen WY., Xu YF, Lv XD, Zhang M., Jiang H., 2018. Polyphyllin Imodulates MALAT1/STAT3 signaling to induce apoptosis in gefitinib -resistant nonsmall cell lung cancer. Toxicol. Appl. Pharmacol. 356, 1–7. doi: 10.1016/j.taap.2018.07.031 30076870

[32] Zhao WW, Liu Y, Feng Q, et al. Ecosystem services for coupled human and environment systems. Progress in Geography, 2018, 37(1): 139–151.

[33] De Groot R, Wilson MA, Boumans R MJ. 2002. A typology for the classification, description and valuation of ecosystem functions, goods and services [J]. Journal of Ecological Economics, 41: 393–408.

[34] Straton A. 2006. A complex systems approach to the value of ecological resources [J]. Journal of Ecological Economics, 56: 402–411.

[35] Hu HB, Liu HY, Hao JF, et al. Effects of urbanization on the spatial hete-rogeneity of watershed ecosystem service value: A case study of Jiuxiang River Watershed in Nanjing City. Journal of Natural Resources, 2011, 26(10): 1715–1725.

[36] Zeng J, Li JF, Yao XW. Spatio -temporal dynamics of ecosystem service value in Wuhan Urban Agglomeration. Chinese Journal of Applied Ecology, 2014, 3(25): 883–891. 24984511

